# Changes in Lower Limb Muscle Activation and Degree of Weight Support according to Types of Cane-Supported Gait in Hemiparetic Stroke Patients

**DOI:** 10.1155/2020/9127610

**Published:** 2020-09-23

**Authors:** Eun Pyeong Choi, Seong Ju Yang, A. Hyun Jung, Hye Su Na, Yeong Ok Kim, Ki Hun Cho

**Affiliations:** Department of Physical Therapy, Korea National University of Transportation, 61 Daehak-ro, Jeungpyeong-gun, Chungbuk 27909, Republic of Korea

## Abstract

This study was aimed at investigating the changes in the degree of weight support loaded on the cane and paretic-side lower limb muscle activation according to the types of cane and cane-supported gait using a weight-support feedback cane (WSFC). Eleven hemiparetic stroke patients were recruited from a local rehabilitation hospital. WSFC can measure the degree of weight support loaded on the cane during cane-supported walking in units of kg, through a force sensor installed inside the handle. This study measured the degree of weight support loaded on the cane and lower limb muscle activation under four conditions: two-point and three-point gait with mono and quadripod canes. In the two-point gait with mono and quadripod canes, subjects were asked to move the WSFC and paretic-side foot forward at the same time and then move the nonparetic-side foot. In the three-point gait with mono and quadripod canes, subjects were asked to first move the WSFC forward, then the paretic-side foot, and finally the nonparetic-side foot. The degree of weight support loaded on the cane was significantly higher in the three-point gait with WSFC than in the two-point gait with WSFC for both mono (*P* = .047) and quadripod canes (*P* = .002). Additionally, the paretic-side lower limb muscle activation during the stance phase was significantly higher in the two-point gait with WSFC than in the three-point gait with WSFC for both mono (*P* = .008 ~ .044) and quadripod canes (*P* = .008 ~ .026). Our results suggest that applying the three-point gait with high cane dependence in the early stages of training for stability and subsequently applying the two-point gait for the enhancement of lower limb muscle activation and training of normal gait pattern could be effective.

## 1. Introduction

Stroke is a typical cerebrovascular disease caused by impaired blood flow [1]. The common symptoms of stroke are spasticity, cognitive impairment, motor and sensory paralysis, and impaired balance and gait functions [2]. Particularly, poststroke hemiparesis induces a decline in motor abilities and asymmetrical movements, thereby leading to a decline in balance and walking abilities, daily living activities, and participation in social activities [3].

In stroke patients, approximately 61-81% of the total body weight is concentrated in the lower limb of the nonparetic side due to hemiparesis [4]. Stroke patients generally have a strong tendency to use the nonparetic lower limb than the paretic side, which makes functional movements such as balance control or walking more difficult [5]. Walking, a complex mechanism generated through the organic relationship of the nervous and the musculoskeletal systems, is an indispensable component of human life [6]. As the recovery of walking ability is a critical factor for the return to independent living [7], it is one of the important goals of stroke rehabilitation [8].

In stroke rehabilitation, walking assistive devices such as crutches, canes, and walkers are used to facilitate balance control ability and to provide stability during walking [9, 10]. In particular, canes help in maintaining the standing posture by assisting the extensor muscles of the hip and back and contribute to enhanced stability during movement [11].

Buurke et al. [12] reported that using a cane during poststroke gait training can contribute to the enhancement of muscle activation in the lower limbs, and another study reported that a cane provides stability by increasing the base of support in stroke patients with an unstable gait pattern [13]. Moreover, it has been reported that cane usage provides stability during the stance phase of gait by decreasing weight-bearing on the paretic-side leg [14]. As such, canes are generally known to be a useful assistive device for stroke patients with reduced functional movement; however, there are also negative reports on cane usage during walking.

Neumann reported that cane usage increases weight-bearing on the nonparetic lower limb by 40%, which interferes with weight-bearing training on the paretic side and results in gait asymmetry and inefficient gait pattern in the longer term [15]. Furthermore, it was reported that the continued and excessive weight-bearing on the nonparetic-side lower limb can cause pain induced by the overuse of the nonparetic-side knee joint and induce a secondary decline in the walking ability [16]. In other words, although cane usage during walking can provide stability in functional movements in hemiparetic stroke patients, indiscriminate use of a cane can be a risk factor of asymmetrical walking.

However, there has been no specific clinical guideline for cane-supported gait in stroke patients to date. Moreover, for gait training in stroke patients, although many types of cane-support gait have been applied, research on canes is mostly focused on exploring the appropriate cane height [17, 18] and the effects of different cane types [19, 20]. In addition, research on the comparison of the paretic-side lower limb muscle activation and the weight load on the cane (cane dependency) according to the methods of cane-supported gait remains insufficient. Thus, this study was aimed at investigating the changes in the degree of weight support loaded on the cane and paretic-side lower limb muscle activation according to the types of cane and cane-supported gait using a weight-support feedback cane (WSFC) that had been designed to quantitatively measure the weight support load on the cane. In this study, the paretic lower limb muscle activation and the load on the cane were measured during two types of cane-supported gait (two-point and three-point gait) using mono and quadripod canes.

## 2. Methods

### 2.1. Subjects

Eleven stroke patients were recruited from a local rehabilitation hospital after attaining a full understanding of the purpose and methods of the research and signing a consent form. The inclusion criteria were as follows: (1) hemiparesis from a single stroke that occurred at least 6 months prior to the time of recruitment, (2) adequate cognition levels to follow simple instructions and understand the content and purpose of the study (Korean version of the Mini-Mental State Examination score ≥ 24 points), (3) ability to walk with a cane (functional ambulation classification, 2–3) [21], and (4) load more than 7% of the body weight on the cane [22]. The exclusion criteria were as follows: (1) musculoskeletal conditions that could potentially affect the ability to walk safely, (2) hemispatial neglect (line bisection test ≥ 12.5 mm), and (3) severe heart disease (heart failure and arrhythmia) or uncontrolled hypertension. To investigate the enrolled subject's functional activity level, we measured the Korean version of the Modified Barthel Index (K-MBI) (activities of daily living performance) [23], Berg Balance Scale (BBS) (balance ability) [24], and Timed Up and Go (TUG) test (gait ability) [25]. The general characteristics of the subjects are shown in Table 1. In this study, the right side was dominant in all subjects.

### 2.2. Procedure

This study applied a cross-sectional design to investigate the changes in lower limb muscle activation and the degree of weight support loaded on a cane according to the types of cane-supported gait in chronic stroke patients. We explained the objective and experimental procedure of the study to all subjects, and they voluntarily signed informed consent forms. Ethical approval for the study was granted by the Korea National University of Transportation (KNUT IRB 2019-15).

We used a weight-support feedback cane (WSFC) to measure the amount of weight loaded on the cane during cane-supported walking of the subject.

**Figure 1:**
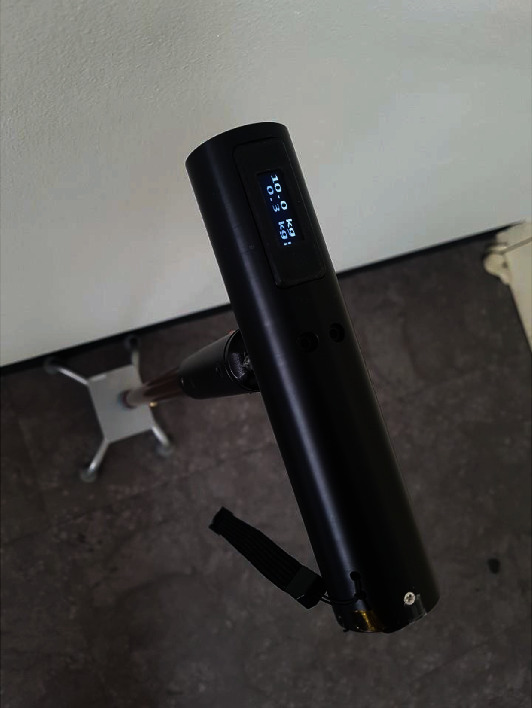
A weight-support feedback cane (WSFC). The WSFC measures cane dependence (degree of weight support loaded on the cane (kg)) during walking. Measurement of the degree of weight support occurs through a load cell located inside the bottom of the cane handle. The degree of weight support is displayed in real time on the cane handle's top display.

**Figure 2:**
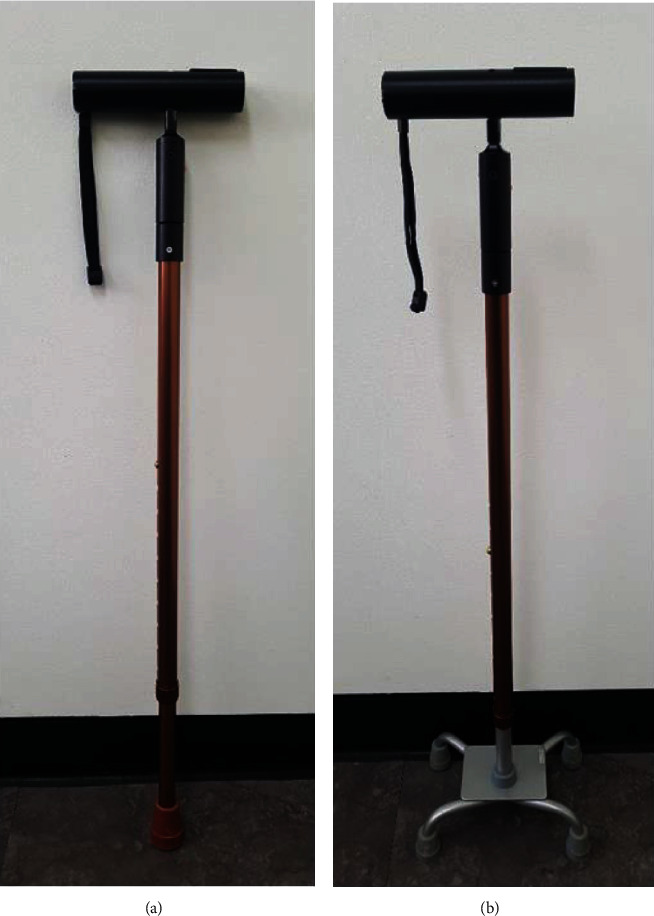
Types of cane-supported gait using a weight-support feedback cane (WSFC). WSFC can be used in a mono cane (a) and in a quadripod cane (b) by switching the cane's legs.

WSFC can measure the amount of force exerted on the cane during cane-supported walking in units of kg, through a force sensor installed inside the handle. The measured cane load was displayed in real time on a monitor located at the top of the handle and computer software connected via Bluetooth (Figure 1). Additionally, WSFC can be used either as a mono cane or a quadripod cane by switching the leg part of the cane (Figure 2). Prior to the experiment, all subjects were asked to walk comfortably for 20 m using the WSFC. We measured the average load on the cane (kg) throughout the 20-meter walk. Subjects with an average load less than 7% of the body weight were excluded from the study.

To investigate the changes in the paretic-side lower limb muscle activation and load on the cane according to the types of cane-supported gait, we performed measurements under four conditions: two-point and three-point gait with WSFC using mono and quadripod canes. All subjects were instructed to hold the WSFC at the height of the greater trochanter using the nonparetic-side hand during measurements.

**Figure 3:**
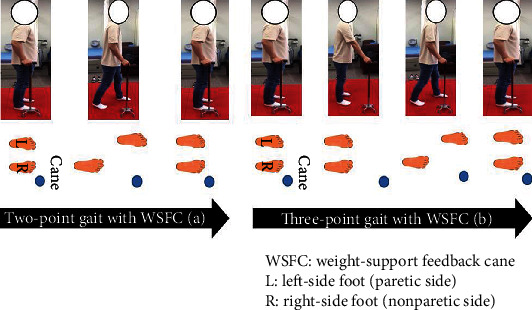
Description of the two-point gait with WSFC (a) and three-point gait with WSFC (b).

For the measurements of the two-point gait with WSFC, subjects were asked to move the WSFC and paretic-side foot forward at the same time and then move the nonparetic-side foot (Figure 3(a)). For the measurements of the three-point gait with WSFC, subjects were asked to first move the WSFC forward, then the paretic-side foot, and, finally, the nonparetic-side foot (Figure 3(b)). The measurements of the two- and three-point gait with WSFC were performed on both mono and quadripod canes. All measurements were taken within the 20-meter walk, and the average load on the WSFC during the 20-meter walk was calculated using the computer software. The paretic-side lower limb muscle activation (rectus femoris, biceps femoris, medial gastrocnemius, tibialis anterior, and gluteus medius) was measured through the wireless surface EMG, while the stance phase of WSFC supported gait. All measurements proceeded after the explication and demonstration of cane-supported walking, and sufficient rest was given in between measurements. Additionally, one assistant was present beside the subject for safety during the experiment.

### 2.3. Measurement

The degree of weight loaded on the cane during two types of cane-supported gait with WSFC was measured using a weight-support feedback cane (WSFC). WSFC measured the degree of force exerted on the cane during cane-supported gait in units of kg, through a force sensor installed inside the handle. Subsequently, the measured cane load was displayed in real time on a monitor located at the top of the handle and computer software.

Additionally, to measure the lower limb muscle activation during two types of cane-supported gait with WSFC, we used a wireless surface EMG (sEMG) (FreeEMG1000, BTS Bioengineering, Milano, Italy) and 3-axis accelerometer (G-Walk, BTS Bioengineering, Italy). The wireless sEMG was used to measure the paretic-side lower limb muscle activation during the stance phase of WSFC gait. Eight wireless sEMG electrodes were attached to the following five major muscle groups of the paretic-side lower limb based on the SENIAM (Surface EMG for Non-Invasive Assessment of Muscles) guideline: rectus femoris, biceps femoris, medial gastrocnemius, tibialis anterior, and gluteus medius [26]. To minimize skin resistance, we removed skin hair at the site of attachment, cleaned the site with alcohol, and attached the electrodes according to the direction of the muscle fibers. Muscle activation data were obtained using an EMG Analyzer v2.9.37.0 (BTS Bioengineering, Milano, Italy). The collected sEMG raw data were band-pass filtered at 20–500 Hz to remove artifact and high-frequency noise. The root mean square (RMS) values were computed over a time constant of 50 ms. Muscle activation data were measured three times and then averaged. To normalize the sEMG signal, all values were set to reference voluntary contraction (RVC) and expressed as %RVC. To obtain %RVC, the EMG signal recorded from the same muscle during standing without movement was used as a reference value.

### 2.4. Statistical Analysis

All statistical analyses were performed using SPSS (version 21.0; IBM Corp., Armonk, NY). Descriptive statistics were used to describe the characteristics of the subjects. The Wilcoxon signed-rank test was used to compare the degree of weight support loaded on a cane and lower limb muscle activation during the two-point and three-point gait with mono and quadripod canes. A significance level of 0.05 was used for all tests.

## 3. Results

A summary of the general characteristics of the 11 subjects who fulfilled the inclusion criteria is shown in Table 1. Tables 2 and 3 show the changes in the degree of weight support loaded on the cane and paretic-side lower limb muscle activation during the stance phase in the two-point and three-point gait with WSFC (mono and quadripod canes).

The degree of weight support loaded on the cane was significantly higher in the three-point gait with WSFC than in the two-point gait with WSFC for both mono (*P* = .047) and quadripod (*P* = .002) canes (Table 2). Additionally, the degree of weight support loaded on the cane was significantly higher in the quadripod cane-supported gait than in the mono cane-supported gait for both of the two-point (*P* = .006) and three-point (*P* = .003) gait with WSFC (Table 2).

The paretic-side lower limb muscle activation during the stance phase was significantly higher in the two-point gait with WSFC than in the three-point gait with WSFC for both mono (rectus femoris: *P* = .043, biceps femoris: *P* = .044, tibialis anterior: *P* = .008, gastrocnemius: *P* = .026, and gluteus medius: *P* = .033) and quadripod (tibialis anterior: *P* = .008, gastrocnemius: *P* = .010, and gluteus medius: *P* = .026; Table 3) canes. However, no significant difference was observed in the comparison of the paretic-side lower limb muscle activation between mono cane- and quadripod cane-supported gait for both of the two-point and three-point gait with WSFC (Table 3).

## 4. Discussion

Hemiparetic stroke patients have increased fall risk due to asymmetric weight-bearing and inefficient gait pattern due to decreased gait symmetry [27]. Therefore, hemiparetic stroke patients receive weight-bearing training on the paretic-side lower limb to reduce asymmetrical weight-bearing, enhance balance control, and induce the formation of symmetrical gait pattern [28]. In clinical settings, canes are primarily used for weight-bearing symmetry training in hemiparetic stroke patients.

Canes are effective in defending against external disturbances during walking [8], and since it secures stability by increasing the base of support [14, 29], it is used primarily in the early stages of gait training. However, excessive dependence on the cane can induce musculoskeletal damage on the nonparetic-side lower limb due to overuse [28] and the formation of asymmetric gait patterns due to nonuse of the paretic-side lower limb. Despite the ongoing controversy over the effectiveness of cane use, there is insufficient research on the quantitative changes in the lower limb weight-bearing capacity due to cane use. The present study thus investigated the changes in weight load on the cane and paretic lower limb muscle activation during the two-point and three-point gait supported by mono and quadripod canes.

According to a previous study of cane-supported gait in hemiparetic stroke patients, the amount of weight-bearing on the paretic-side lower limb decreases as cane dependency increases, whereas the amount of paretic-side lower limb weight-bearing increases as cane dependence decreases [30]. Furthermore, the increase in the amount of paretic-side lower limb weight-bearing due to decreased cane dependence directly contributes to the increase in paretic-side lower limb muscle activation [12]. In particular, the stable maintenance of the single-leg stance during the gait cycle requires concurrent contractions of the lower limb muscles [31].

Our results showed that the degree of weight support loaded on a cane was higher in the three-point gait with WSFC than in the two-point gait with WSFC for both mono and quadripod canes (Table 1). In contrast, the paretic-side lower limb muscle activation during the stance phase was higher in the two-point gait with WSFC than in the three-point gait with WSFC for both the mono and quadripod canes (Table 2).

Although canes can improve stability or weight transfer during walking, improper use of the cane or application of an inappropriate walking pattern may directly increase fall risk [32]. In cane-supported gait, the cane can interfere with lower limb movements during walking [33]. In addition, lifting and advancing the cane can lead to instability in biomechanical forces, and balance may be disrupted by the need to focus on the cane control [34]. Thus, appropriate education for proper cane use from a medical professional is important to increase the efficiency of walking and reduce fall risk [35]. The results of this study suggest that for gait training in hemiplegia, the two-point gait may be more effective than the three-point gait in activating the paretic-side lower limb muscle during the stance phase of the gait cycle. Furthermore, the three-point gait, which has higher cane dependence, may be more effective than the two-point gait in terms of stability. Foot and cane sequence determines the way of cane-supported gait patterns, and each cane-supported gait pattern depends on the subject's ability to tolerate full load on each leg and to maintain balance [32, 36]. The most important thing is that the two-point gait requires a more balanced posture than the three-point gait, but it simulates a gait pattern closer to normal [32]. Therefore, in hemiparetic stroke gait training, the types of cane-supported gait should be considered carefully. In particular, applying the three-point gait with high cane dependence in the early stages of training for stability and subsequently applying the two-point gait for the enhancement of lower limb muscle activation and training of normal gait pattern could be effective.

Another analysis of this study is a comparison of the lower limb muscle activation according to the use of two types of canes. Canes are made of different types of legs to suit user needs and preferences [37], and in clinical settings, two types of canes such as mono (single leg) and quadripod (four legs) canes are commonly used for the symmetrical weight-bearing and gait training in hemiparetic stroke patients. According to a previous study, a mono cane can support approximately one-quarter of a user's body weight, whereas a quadripod cane can support almost half of the user's body weight [32]. In other words, the mono cane supports less weight than a quadripod cane, and this can contribute to the paretic-side lower limb muscle activation during maintaining the standing posture and walking. Based on the results of previous studies [30, 32], we speculated that the paretic-side lower limb muscle activation would be higher in the mono cane-supported gait than in the quadripod cane-supported gait. Interestingly, such a trend was observed in this study, however, not statistically significant (Table 2). In the present study, although we proposed a clinical protocol based on the differences between the types of gait pattern during cane-supported gait training, we have not explored other factors that can affect the lower limb muscle activation during cane-supported walking such as environmental factors and the level of functional activity. In particular, since the extent of the lesion and the dominant side were not considered during the selection process, it is possible that these factors influenced the results of the study. Moreover, this study has a small sample size, and the study population was limited to only high-functioning chronic stroke patients (MBI: 60.72 points, BBS: 36.81 points); therefore, these factors may also have influenced the comparative results of the lower limb muscle activation according to the use of two types of canes. Future studies with large cohorts are warranted to analyze the lower limb muscle activation according to the types of cane and cane-supported gait.

## 5. Conclusions

This study was aimed at investigating the changes in the amount of weight support loaded on the cane and paretic-side lower limb muscle activation according to the types of cane and cane-supported gait using a weight-support feedback cane (WSFC) that had been designed to quantitatively measure the weight support load on the cane. The results of this study showed that applying the three-point gait with a high cane dependence in the early stages of training for stability and subsequently applying the two-point gait for the enhancement of the lower limb muscle activation could be effective. Therefore, in hemiparetic stroke gait training, the selection of the types of cane-supported gait and education for proper cane use should be considered carefully.

## Figures and Tables

**Table 1:** General characteristic of the subjects.

Parameters	Male (*n* = 8)	Female (*n* = 3)	Overall (*n* = 11)
Paretic side			
Left/right	3/5	0/3	3/8
Etiology			
Infarction/hemorrhage	7/1	2/1	9/2
Age (years)	49.37 ± 12.17	53.33 ± 9.45	50.45 ± 11.18
Height (cm)	172.62 ± 8.17	166 ± 6.08	170.81 ± 7.98
Weight (kg)	71.62 ± 8.66	71.66 ± 10.4	71.63 ± 8.61
Brunnstrom stage (2/3/4)	5/3/0	0/2/1	5/5/1
MAS (1/1+/2)	1/4/3	1/0/2	2/4/5
Onset duration (months)	13.25 ± 8.2	16.66 ± 6.02	14.18 ± 7.54
MMSE-K (scores)	29.25 ± 0.88	30	29.45 ± 0.82
K-MBI (scores)	63.25 ± 14.38	54 ± 12.28	60.72 ± 13.92
FAC (2/3)	5/3	2/1	7/4
BBS (scores)	37.25 ± 5.44	35.66 ± 8.14	36.81 ± 5.87
TUG (sec)	46.32 ± 21.95	59.63 ± 22.89	49.95 ± 21.92

Values are expressed as mean ± SD. MAS: Modified Ashworth Scale; MMSE-K: Mini-Mental State Examination-Korean; K-MBI: Korean version of the Modified Barthel Index; FAC: Functional Ambulation Category; BBS: Berg Balance Scale; TUG: Timed Up and Go test; cm: centimeter; kg: kilogram; sec: seconds.

**Table 2:** Changes in the degree of weight support loaded on a cane in the two- and three-point gait with mono and quadripod canes (*n* = 11).

Parameters (kg)	Two-point gait with WSFC	Three-point gait with WSFC	*z* (*P* values)
DWS loaded on mono cane	7.23 ± 3.51	7.75 ± 3.22	-2.011 (.047)^∗^
DWS loaded on quadripod cane	8.14 ± 4.32	9.04 ± 4.91	-3.114 (.002)^∗^
*z* (*P* values)	-2.985 (.006)^∗^	-3.025 (.003)^∗^	

Values are expressed as mean ± SD. kg: kilogram; WSFC: weight-support feedback cane; DWS: degree of weight support. ^∗^*P* < .05.

**Table 3:** Changes in paretic-side lower limb muscle activation in the stance phase according to the types of cane and cane-supported gait (*n* = 11).

Parameters (%RVC)		Two-point gait with WSFC	Three-point gait with WSFC	*z* (*P*) values
RF	Mono cane-supported gait	187.72 ± 87.73	158.98 ± 77.28	-1.956 (.043)^∗^
	Quadripod cane-supported gait	179.82 ± 102.46	160.96 ± 82.91	-1.156 (.248)
	*z* (*P* values)	-0.321 (.534)	-0.178 (.859)	

BF	Mono cane-supported gait	152.32 ± 78.87	122.56 ± 49.96	-1.886 (.044)^∗^
	Quadripod cane-supported gait	142.74 ± 66.09	123.60 ± 39.45	-1.600 (.110)
	*z* (*P* values)	-1.172 (.241)	-0.561 (.575)	

TA	Mono cane-supported gait	539.87 ± 312.38	409.76 ± 241.86	-2.667 (.008)^∗^
	Quadripod cane-supported gait	523.66 ± 267.03	374.61 ± 218.21	-2.667 (.008)^∗^
	*z* (*P* values)	-0.356 (.722)	-0.800 (.424)	

GCM-M	Mono cane-supported gait	141.24 ± 98.30	111.13 ± 65.29	-2.223 (.026)^∗^
	Quadripod cane-supported gait	137.92 ± 86.41	115.87 ± 67.93	-2.578 (.010)^∗^
	*z* (*P* values)	-0.445 (.657)	-1.067 (.286)	

GM	Mono cane-supported gait	149.85 ± 86.73	108.54 ± 37.50	-2.134 (.033)^∗^
	Quadripod cane-supported gait	116.81 ± 44.64	100.49 ± 38.65	-2.223 (.026)^∗^
	*z* (*P* values)	-1.867 (.062)	-1.067 (.286)	

Values are expressed as mean ± SD. RVC: reference voluntary contraction; WSFC: weight-support feedback cane; RF: rectus femoris; BF: biceps femoris; TA: tibialis anterior; GCM-M: gastrocnemius (medial part); GM: gluteus medius. ^∗^*P* < .05.

## Data Availability

The datasets used and analyzed during the current study are available from the corresponding author on reasonable request.
